# Health Disparities Among American Indians/Alaska Natives — Arizona, 2017

**DOI:** 10.15585/mmwr.mm6747a4

**Published:** 2018-11-30

**Authors:** Monique Adakai, Michelle Sandoval-Rosario, Fang Xu, Teresa Aseret-Manygoats, Michael Allison, Kurt J. Greenlund, Kamil E. Barbour

**Affiliations:** ^1^Arizona Department of Health Services; ^2^Division of Population Health, National Center for Chronic Disease Prevention and Health Promotion, CDC.

Compared with other racial/ethnic groups, American Indians/Alaska Natives (AI/AN) have a lower life expectancy, lower quality of life, and are disproportionately affected by many chronic conditions (*1*,*2*). Arizona has the third largest population of AI/AN in the United States (approximately 266,000 in 2017), and is home to 22 federally recognized American Indian tribal nations.* The small AI/AN sample size in previous Behavioral Risk Factor Surveillance System (BRFSS) surveys has presented analytic challenges in making statistical inferences about this population. To identify health disparities among AI/AN living in Arizona, the Arizona Department of Health Services (ADHS) and CDC analyzed data from the 2017 BRFSS survey, for which AI/AN were oversampled. Compared with whites, AI/AN had significantly higher prevalences of sugar-sweetened beverage consumption (33.0% versus 26.8%), being overweight or having obesity (76.7% versus 63.2%), diabetes (21.4% versus 8.0%), high blood pressure (32.9% versus 27.6%), report of fair or poor health status (28.7% versus 16.3%), and leisure-time physical inactivity during the past month (31.1% versus 23.0%). AI/AN also reported a lower prevalence of having a personal doctor or health care provider (63.1%) than did whites (72.8%). This report highlights the need to enhance surveillance measures at the local, state, and national levels and can inform interventions centered on confronting social inequities, developing culturally competent prevention strategies, and facilitating access to care to improve population health and work toward health equity.

BRFSS^†^ is a telephone (landline and cellular) survey conducted annually in all 50 states, the District of Columbia, and U.S. territories to collect information on health-related behavioral risk factors, health care access, and chronic conditions among noninstitutionalized U.S. adults aged ≥18 years. To increase sample size and representation of AI/AN, the U.S. Department of Health and Human Services Office of Minority Health collaborated with CDC to oversample AI/AN in 11 states to improve understanding of their health status.^§^ Data from the 2017 Arizona BRFSS (15,004) were used to examine the prevalence of selected sociodemographic characteristics, lifestyle health-related behaviors, and chronic conditions among AI/AN, compared with prevalences among whites and other races. In 2017, Arizona BRFSS landline and cellular response rates were 52.3% and 79.6%, respectively. Race was categorized as white or AI/AN according to the BRFSS variable denoting preferred race category^¶^ based on the response to the following question: “Which one or more of the following would you say is your race?” The preferred race category was selected to avoid missing or excluding persons self-identifying as AI/AN, regardless of Hispanic ethnicity. Other races** (others) were defined as any race category apart from white or AI/AN. Age-adjusted prevalences standardized to the projected 2000 U.S. population^††^ with 95% confidence intervals (CIs) were calculated for sociodemographic characteristics (sex, marital status, education level, income, and employment status), access to care^§§^ (health care coverage and having a personal doctor or health care provider), lifestyle indicators^¶¶^ (current smoking, current smokeless tobacco use, binge drinking, consumption of sugar-sweetened beverages, and physical inactivity), and health status and chronic conditions*** (frequent mental distress, being overweight or having obesity, and doctor-diagnosed coronary heart disease, asthma, chronic obstructive pulmonary disease, diabetes, arthritis, high blood pressure, high blood cholesterol, and depression). Group differences were assessed with pairwise tests (AI/AN versus whites and AI/AN versus others) with statistical significance defined as p<0.05. Only statistically significant results are presented. Statistical software was used to account for survey weights and complex survey design.

Among all 15,004 respondents, 766 (5.1%) identified their race as AI/AN, 12,472 (76.3%) as white, and 1,766 (18.6%) as other. Among AI/AN, the prevalences of having less than a high school diploma (23.2%), reporting <$15,000 annual income (22.8%), and reporting unemployment (11.6%) were higher than those among whites (11.8%, 6.7%, and 5.9%, respectively) (Table 1). The prevalence of having health care coverage was higher among AI/AN (74.1%) than that among whites (71.7%) and others (65.3%), but the prevalence among AI/AN of having a personal doctor or health care provider (63.1%) was lower than that among whites (72.8%) and others (67.6%) (Table 1). The prevalences among AI/AN reporting fair or poor health status (28.7%), being overweight or having obesity (76.7%), and having diabetes (21.4%) were higher than those among whites (16.3%, 63.2%, and 8.0%, respectively) and others (23.6%, 65.9%, and 13.1%, respectively) (Table 2). In addition, among AI/AN, the prevalences of leisure-time physical inactivity (31.1%), daily sugar-sweetened beverage consumption (33.0%) and high blood pressure (32.9%) were higher than those among whites (23.0%, 26.8%, and 27.6%, respectively) (Table 2).

**TABLE 1:** Age-adjusted* weighted prevalence of sociodemographic characteristics and health care access among American Indians/Alaska Natives, whites, and adults aged ≥18 years with other race (total estimated population = 5,192,000) — Behavioral Risk Factor Surveillance System, Arizona, 2017

Characteristic	American Indians/Alaska Natives^†^			Whites^†^			Others^†^		
	n = 766; weighted % = 5.1			n = 12,472; weighted % = 76.3			n = 1,766; weighted % = 18.6		
	Unweighted sample size, no.	Estimated population, no.	Weighted % (95% CI)	Unweighted sample size, no.	Estimated population, no.	Weighted % (95% CI)	Unweighted sample size, no.	Estimated population, no.	Weighted % (95% CI)
**Age group (yrs)**									
18–24	64	39,600	14.9 (11.1–18.6)	559	446,600	11.3 (10.3–12.2)	192	188,000	19.5 (16.8–22.1)
25–44	252	111,000	41.7 (37.0–46.5)	2,233	1,211,100	30.6 (29.4–31.7)	581	401,000	41.5 (38.6–44.4)
45–64	315	84,400	31.7 (27.5–35.9)	4,224	1,254,000	31.7 (30.7–32.6)	632	277,000	28.7 (26.3–31.1)
≥65	135	31,000	11.7 (9.1–14.3)	5,456	1,049,000	26.5 (25.7–27.3)	361	100,000	10.3 (9.0–11.6)
**Sex**									
Male	328	141,000	52.9 (48.2–57.5)	5,547	1,904,000	48.6 (47.3–49.9)	871	500,000	52.1 (49.3–54.9)
Female	435	125,000	47.2 (42.5–51.8)	6,910	2,052,000	51.4 (50.1–52.7)	892	465,000	47.9 (45.2–50.7)
**Marital status**									
Married^††,§§^	255	82,500	31.9 (27.8–36.1)	6,768	2,079,000	51.4 (50.2–52.6)	804	421,000	46.8 (44.1–49.4)
Divorced/Widowed/Separated^††,§§^	209	57,300	23.5 (20.1–27.0)	3,697	879,800	19.6 (18.7–20.4)	439	161,000	19.2 (17.3–21.1)
Never married/Member of an unmarried couple^††,§§^	302	126,400	44.5 (40.6–48.4)	2,007	1,002,000	29.0 (28.0–30.1)	523	384,000	34.1 (31.8–36.4)
**Education**									
Less than high school^††^	101	59,100	23.2 (18.6–27.7)	588	450,400	11.8 (10.7–12.9)	226	202,100	22.4 (19.7–25.0)
High school/GED^††,§§^	257	83,300	30.8 (26.8–34.8)	2,739	951,700	24.1 (23.1–25.2)	475	265,000	26.1 (23.8–28.4)
College/Technical school or higher^††,§§^	403	121,600	45.2 (40.6–49.7)	9,111	2,549,000	63.9 (62.6–65.2)	1,054	490,800	50.7 (47.9–53.5)
**Annual income**									
<$15,000^††,§§^	178	60,000	22.8 (18.7–26.9)	755	261,800	6.7 (6.0–7.4)	173	93,600	10.2 (8.5–11.8)
$15,000–$34,999	222	68,200	26.4 (22.5–30.3)	2,618	882,400	21.9 (20.8–23.0)	457	263,200	27.1 (24.6–29.6)
$35,000–$74,999^††^	154	51,200	19.3 (15.7–22.9)	3,347	1,042,200	26.5 (25.3–27.6)	388	204,400	20.8 (18.6–22.9)
≥$75,000^††,§§^	76	27,900	10.3 (7.4–13.2)	3,526	1,078,600	28.3 (27.2–29.4)	333	174,200	18.1 (16.0–20.2)
Unknown/Refused	127	55,000	19.8 (15.9–23.7)	2,169	671,700	16.1 (15.1–17.1)	400	221,000	22.9 (20.5–25.2)
**Employment status** ^§^									
Employed/Self-employed^††,§§^	350	128,600	45.8 (41.5–50.2)	5,435	2,093,800	58.5 (57.3–59.7)	947	576,700	57.3 (54.9–59.7)
Unemployed^††,§§^	82	32,400	11.6 (8.5–14.7)	475	209,600	5.9 (5.2–6.6)	101	59,300	5.6 (4.4–6.8)
Unable to work	219	66,300	28.1 (24.5–31.7)	5,750	1,371,500	28.5 (27.5–29.5)	547	249,300	28.2 (26.0–30.3)
Other^††,§§^	105	36,500	13.5 (10.1–17.0)	731	251,000	6.2 (5.6–6.7)	147	63,000	7.2 (5.9–8.5)
**Have health care coverage** ^¶,††,§§^	553	206,600	74.1 (71.3–76.9)	6,153	2,483,800	71.7 (70.7–72.6)	1,099	658,600	65.3 (63.0–67.5)
Have a personal doctor or health care provider*^*,††,§§^	504	163,800	63.1 (58.7–67.4)	10,399	3,001,800	72.8 (71.5–74.0)	1,269	617,000	67.6 (65.1–70.2)
**Total estimated population**	**266,000**			**3,960,000**			**966,000**		

**Abbreviations:** CI = confidence interval; GED = general educational development certificate.

* https://www.cdc.gov/nchs/data/statnt/statnt20.pdf.

^†^ Respondents were identified as American Indians/Alaska Natives, whites or others according to the BRFSS variable denoting preferred race category, a calculated race variable. It does not specify Hispanic ethnicity. Response options available to respondents included White, Black or African American, American Indian or Alaska Native, Asian, Pacific Islander. Others were defined as respondents selecting any of the other race categories including Black or African American, Asian, Native Hawaiian or other Pacific Islander, Other race, No preferred race. Respondents who did not select a single race were defined as “Don’t know/Not sure” or “Refused” and were coded as missing and not included in the analysis.

^§^ Employment status was defined by respondents who answered “Are you currently..? Employed for wages, self-employed, out of work for 1 year or more, out of work for less than 1 year, a homemaker, a student, retired, or unable to work.” “Employed” was defined according to an affirmative response to employed or self-employed. “Unemployed” was defined according to an affirmative response to out of work for 1 year or more or out of work for less than 1 year. Other was defined according to an affirmative response to any of the following categories: a homemaker, a student, and retired.

^¶^ Health care coverage was defined by affirmative responses by respondents aged 18–64 years to the following question: “Do you have any kind of health care coverage, including health insurance, prepaid plans such as HMOs, or government plans such as Medicare, or Indian Health Service?”

** Have access to a health care provider was defined by a response of “yes,” “only one,” or “more than one” to the following question: “Do you have one person you think of as your personal doctor or health care provider?”

^††^ Characteristic differed significantly between American Indians/Alaska Natives and whites (p<0.05).

^§§^ Characteristic differed significantly between American Indians/Alaska Natives and others (p<0.05).

**TABLE 2:** Age-adjusted* weighted prevalence of lifestyle health-related behaviors and chronic conditions among American Indians/Alaska Natives, whites, and adults aged ≥18 years with other race (total estimated population = 5,192,000) — Behavioral Risk Factor Surveillance System, Arizona, 2017

Characteristic	American Indians/Alaska Natives^†^			Whites^†^			Others^†^		
	n = 766; weighted % = 5.1			n = 12,472; weighted % = 76.3			n = 1,766; weighted % = 18.6		
	Unweighted sample size, no.	Estimated population, no.	Weighted % (95% CI)	Unweighted sample size, no.	Estimated population, no.	Weighted % (95% CI)	Unweighted sample size, no.	Estimated population, no.	Weighted % (95% CI)
**Health-related behaviors** ^§^									
Current smoker	89	38,900	15.7 (11.9–19.5)	1,596	601,700	16.7 (15.6–17.7)	240	128,500	14.8 (12.7–16.9)
Current smokeless tobacco user**	47	12,400	4.8 (2.9–6.7)	275	109,600	3.2 (2.7–3.7)	36	18,900	2.2 (1.3–3.0)
Binge drinking**	81	41,700	17.6 (13.6–21.6)	1,310	553,000	16.9 (15.8–18.0)	207	126,400	14.0 (12.0–16.1)
Sugar-sweetened beverage ≥1 time per day^††^	178	65,100	33.0 (27.9–38.1)	2,031	793,500	26.8 (25.4–28.2)	390	224,700	31.4 (28.4–34.4)
Leisure-time physical inactivity^††^	186	66,900	31.1 (26.4–35.8)	2,804	873,900	23.0 (21.9–24.1)	398	212,700	27.6 (24.8–30.3)
**Health status and chronic conditions** ^¶^									
Fair/Poor health status**^,††^	219	75,000	28.7 (24.4–33.0)	2,195	686,000	16.3 (15.4–17.2)	398	214,400	23.6 (21.2–26.0)
Frequent mental distress	103	40,700	15.1 (11.5–18.8)	1,291	474,700	12.8 (11.9–13.7)	212	115,900	12.1 (10.3–13.9)
Asthma	88	30,000	11.8 (8.5–15.1)	1,271	408,100	10.5 (9.7–11.3)	158	75,800	7.9 (6.5–9.3)
Overweight or having obesity**^,††^	519	183,800	76.7 (72.6–80.7)	7,394	2,323,900	63.2 (61.9–64.4)	1,062	546,200	65.9 (63.0–68.7)
Coronary heart disease	50	13,700	5.8 (3.9–7.8)	1,143	263,100	5.2 (4.8–5.7)	103	34,900	4.7 (3.6–5.8)
Chronic obstructive pulmonary disease	41	12,700	5.3 (3.3–7.3)	1,119	289,800	6.4 (5.8–6.9)	93	31,000	3.9 (3.0–4.7)
Diabetes**^,††^	179	52,800	21.4 (18.0–24.7)	1,507	381,800	8.0 (7.5–8.6)	256	103,300	13.1 (11.5–14.8)
Arthritis	169	52,200	21.5 (17.8–25.2)	4,347	1,076,700	23.4 (22.5–24.3)	379	140,700	17.4 (15.6–19.2)
High blood pressure^††^	262	81,000	32.9 (29.1–36.8)	4,970	1,264,200	27.6 (26.6–28.5)	595	246,800	29.6 (27.4–31.9)
High cholesterol	464	164,200	74.1 (69.6–78.5)	6,626	2,179,800	69.6 (68.4–70.8)	1,038	588,100	72.0 (69.6–74.5)
Depression**	121	45,700	17.2 (13.6–20.8)	2,387	797,600	20.7 (19.6–21.7)	280	132,100	13.7 (11.9–15.5)
**Total estimated population**	**266,000**			**3,960,000**			**966,000**		

**Abbreviation**: CI = confidence interval.

* https://www.cdc.gov/nchs/data/statnt/statnt20.pdf.

^†^ Respondents were identified as American Indians/Alaska Native (AI/AN), white, or others according to the BRFSS variable denoting preferred race category, a calculated race variable. It does not specify Hispanic ethnicity. Response options available to respondents included White, Black or African American, American Indian or Alaska Native, Asian, Pacific Islander. Others were defined as respondents selecting any of the other race categories including Black or African American, Asian, Native Hawaiian or other Pacific Islander, Other race, No preferred race. Respondents who did not select a single race were defined as “Don’t know/Not sure” or “Refused” and were coded as missing and not included in the analysis.

^§^ Current smoking was defined as reporting smoking ≥100 cigarettes during one’s lifetime and currently smoking every day or some days. Current smokeless tobacco use was defined as a response of “Every day” or “Some days” to the following question: “Do you currently use chewing tobacco, snuff, or snus every day, or not at all?” Binge drinking was defined as having ≥5 drinks on one occasion (men) or ≥4 drinks on one occasion (women). In 2017, the BRFSS included an optional module with two sugar-sweetened beverage intake questions: 1) “During the past 30 days, how often did you drink regular soda or pop that contains sugar? Do not include diet soda or diet pop.” and 2) “During the past 30 days, how often did you drink sugar-sweetened fruit drinks (such as Kool-Aid and lemonade), sweet tea, and sports or energy drinks (such as Gatorade and Red Bull)? Do not include 100% fruit juice, diet drinks, or artificially sweetened drinks.” Respondents answered number of times per month, week, or day, and responses were converted to daily intake. To calculate daily intake frequency, both questions were summed and categorized as none, >0 to <1, and ≥1 time per day. Consumption of sugar-sweetened beverage was defined as consumption ≥1 time per day (https://www.cdc.gov/brfss/data_documentation/pdf/brfss_ssb-userguide.pdf). Physical inactivity was defined according to a non-confirmatory response to the following question: “During the past month, other than your regular job, did you participate in any physical activities or exercises such as running, calisthenics, golf, gardening, or walking for exercise?”

^¶^ Respondents rated their general health as being excellent, very good, good, fair, or poor. The responses were then categorized into two groups: 1) those who reported that their health was excellent, very good, or good and 2) those who reported that their health was fair or poor. Fair or poor health status was defined as a report of fair or poor health. All respondents were asked to determine how many days during the past 30 days their mental health status (e.g., stress, depression, and problems with emotions) was not good. The respondents were divided into two groups: those who reported frequent mental distress (≥14 mentally unhealthy days during the past 30 days) and those who reported no frequent mental distress (<14 mentally unhealthy days during the past 30 days). Frequent mental distress was defined as a report of ≥14 mentally unhealthy days during the past 30 days. Overweight or having obesity was defined as a body mass index ≥25 kg/m^2^, or ≥30 kg/m^2^, respectively, calculated from self-reported weight and height. Coronary heart disease was defined as having ever been told by a doctor, nurse, or other health care professional that the respondent had a heart attack (myocardial infarction) or angina. Asthma was defined as having ever been told by a doctor, nurse, or other health care professional that the respondent had asthma and still had it at the time of survey participation. Chronic obstructive pulmonary disease was defined as having ever been told by a doctor, nurse, or other health care professional that the respondent had chronic obstructive pulmonary disease, emphysema, or chronic bronchitis. Diabetes was defined as having ever been told by a doctor, nurse, or other health care professional that the respondent had diabetes, excluding gestational diabetes, prediabetes, or borderline diabetes. Arthritis was defined as having ever been told by a doctor, nurse, or other health care professional that the respondent had some form of arthritis, rheumatoid arthritis, gout, lupus, or fibromyalgia. High blood pressure was defined as having ever been told by a doctor, nurse, or other health care professional the respondent had high blood pressure. High blood cholesterol was defined as having ever been told by a doctor, nurse, or other health care professional that the respondent’s blood cholesterol was high. Depression was defined as having ever been told by a doctor, nurse, or other health care professional that the respondent had a depressive disorder, which includes depression, major depression, dysthymia, or minor depression.

** Significant association between American Indians/Alaska Natives and others (p<0.05).

^††^ Significant association between American Indians/Alaska Natives and whites (p<0.05).

## Discussion

BRFSS estimates in this report were based on an oversampling of AI/AN in Arizona, to obtain data to inform strategies for mitigating health disparities among AI/AN (*3*,*4*). Consistent with other findings (*5*), these data indicate lower levels of educational attainment and income, and higher levels of unemployment among AI/AN, compared with those among whites and others, indicative of the disadvantages faced by AI/AN. Addressing these issues is important to decreasing the high prevalence and incidence of chronic conditions among AI/AN (*6*).

In 2017, the prevalence of self-reported health care coverage was higher among AI/AN than among whites and others. An example of health care coverage listed in the BRFSS question is Indian Health Service (IHS), which is a health care system that provides clinical, behavioral, and limited specialty health care services to enrolled members of federally recognized AI/AN tribes.^†††^ With IHS, access to health care services is only available at federal hospitals and clinics operated or funded by IHS and might not ensure that AI/AN have ready access to health interventions or coverage to see non-IHS providers. Thus, although respondents reported having health care coverage, many might not be able to access care beyond IHS facilities. Review of the BRFSS question on health care coverage might be necessary to distinguish between respondents reporting Medicare, Medicaid, IHS, Veterans Administration, private health insurance, or being uninsured.

Prevalence of having a personal doctor or health care provider was lower among AI/AN than among whites and others. Historically, IHS facilities are located in geographically isolated areas on reservations (*6*). As the AI/AN population has become younger and more racially diverse, larger numbers of AI/AN are residing in cities, limiting continuity of care through IHS (*5*,*7*) and possibly the ability of AI/AN to obtain and retain a personal doctor or health care provider. Studies have highlighted additional barriers preventing AI/AN from accessing providers, including long wait times; travel time to an IHS facility; and lack of or limited access to transportation, culturally and linguistically appropriate providers, a full range of services, preventive care, screening, and early treatment for health conditions (*3*,*8*,*9*).

In 2015, the top five leading causes of death for AI/AN in Arizona were unintentional injury, cancer, coronary heart disease, chronic liver disease and cirrhosis, and diabetes (*10*). When compared with the entire U.S. population, diabetes and chronic liver disease and cirrhosis are more common causes of death among the AI/AN population. Population-level behavioral and policy interventions are needed to reduce disparities in diabetes and chronic liver disease and cirrhosis mortality in the AI/AN population. These current analyses indicated a higher prevalence of sugar-sweetened beverage intake, leisure-time physical inactivity, being overweight or having obesity, and having diabetes or high blood pressure among AI/AN compared with whites in Arizona. Population-specific data on these indicators is crucial to formulating data-informed strategic plans and priority setting at ADHS. The Arizona Health Improvement Plan^§§§^ provides a structure to link networks of partners to align resources and programs to improve the health of persons and communities across Arizona using evidence-based preventive health strategies.

These findings identified a number of health disparities among AI/AN in Arizona, which will require a concerted effort and culturally tailored public health approaches to address. ADHS’s Native American liaison serves as a link between ADHS and tribal communities, tribal health offices, urban Indian health programs, IHS area offices, and other local, state, and federal organizations. Moreover, CDC funding mechanisms (e.g., “Tribal Practices for Wellness in Indian Country”^¶¶¶^ and “Good Health and Wellness in Indian Country”****) help to identify culturally tailored public health approaches to reduce risk factors for chronic diseases. Support for culturally competent public health approaches over time could potentially facilitate the elimination of health disparities.

The findings in this report are subject to at least seven limitations. First, BRFSS information collected is self-reported; therefore, study findings might be subject to recall and social desirability biases. Second, the prevalence of conditions represents only diagnosed disease, not underdiagnosed disease, which is an important factor and might be different among groups. Third, although weighting methods are used to account for nonresponse bias and differential probability of selection in BRFSS data, bias might still exist. Fourth, results presented for AI/AN are intended to be representative of all tribes in Arizona; even so, results do not record variation among different tribal groups in Arizona or other tribes across the United States. Fifth, place of residence (urban, suburban, rural, or reservation) was not elucidated in BRFSS data but might influence the degree to which health disparities or risk behaviors affect certain groups. Sixth, publicly available state-based survey weights from the Arizona BRFSS data set were used, and reported results might slightly differ for the AI/AN population if more precise population-specific survey weights are used. Finally, because this is a cross-sectional study, causality cannot be inferred.

Characterizing health disparities adds to the understanding of AI/AN population health. Enhanced surveillance measures at the local, state, and national level can increase awareness about health challenges faced by this population, which will be instrumental to improving health and working toward health equity. Nonetheless, challenges associated with confronting social inequities, effectively working through cultural differences, increasing health literacy within the AI/AN population, and eliminating roadblocks that limit access to care will need to be overcome (*6*–*9*). In addition, tribal, state, and federal entities need to work together to address disparities. Documenting characteristics contributing to the health of AI/AN can better equip health professionals to identify priorities and culturally and linguistically appropriate interventions to improve health and decrease health disparities.

**Summary**

**What is already known about this topic?**

American Indians/Alaska Natives (AI/AN) have a lower life expectancy, a lower quality of life, and a higher prevalence of many chronic conditions.

**What is added by this report?**

Analysis of 2017 Behavioral Risk Factor Surveillance System data from Arizona found significantly higher prevalences of sugar-sweetened beverage consumption, being overweight or having obesity, diabetes, hypertension, fair or poor health status, and leisure-time physical inactivity and a lower prevalence of having a personal doctor among AI/AN compared to whites.

**What are the implications for public health practice?**

Culturally tailored public health approaches to reducing risk factors and chronic diseases among AI/AN are needed. Improved surveillance can better equip health professionals to identify priorities and implement interventions to improve health and reduce disparities among AI/AN.

## Footnotes

* https://pub.azdhs.gov/health-stats/report/hspam/2016/index.php.

† https://www.cdc.gov/brfss/index.html.

§ https://minorityhealth.hhs.gov/omh/browse.aspx?lvl = 2&lvlid = 89.

¶ Respondents were identified as AI/AN, whites, or others according to the BRFSS variable denoting preferred race category, a calculated race variable. Response options available to respondents included White, Black or African American, American Indian or Alaska Native, Asian, Pacific Islander. Respondents who did not select a single race were defined as “Don’t know/Not sure” or “Refused” and were coded as missing and not included in the analysis.

** Others were defined as respondents selecting any race category other than white or AI/AN, including black or African American, Asian, Native Hawaiian or other Pacific Islander, Other race, or No preferred race, regardless of Hispanic ethnicity.

†† https://www.cdc.gov/nchs/data/statnt/statnt20.pdf.

§§ Health care coverage was defined among respondents aged 18–64 years who answered “yes” to the following question: “Do you have any kind of health care coverage, including health insurance, prepaid plans such as HMOs, or government plans such as Medicare, or Indian Health Service?” Having access to a health care provider was defined by responses of “yes,” “only one” or “more than one” to the following question: “Do you have one person you think of as your personal doctor or health care provider?”

¶¶ Current smoking was defined as reporting smoking ≥100 cigarettes during one’s lifetime and currently smoking every day or some days. Current smokeless tobacco use was defined as a response of “Every day” or “Some days” to the following question: “Do you currently use chewing tobacco, snuff, or snus every day, or not at all?” Binge drinking was defined as having ≥5 drinks on one occasion (men) or ≥4 drinks on one occasion (women). In 2017, the BRFSS included an optional module with two sugar-sweetened beverage intake questions: 1) “During the past 30 days, how often did you drink regular soda or pop that contains sugar? Do not include diet soda or diet pop.” and 2) “During the past 30 days, how often did you drink sugar-sweetened fruit drinks (such as Kool-Aid and lemonade), sweet tea, and sports or energy drinks (such as Gatorade and Red Bull)? Do not include 100% fruit juice, diet drinks, or artificially sweetened drinks.” Respondents answered number of times per month, week, or day, and responses were converted to daily intake. To calculate daily intake frequency, both questions were summed and categorized as none, >0 to <1, and ≥1 time per day. Consumption of sugar-sweetened beverage was defined as consumption ≥1 time per day (https://www.cdc.gov/brfss/data_documentation/pdf/brfss_ssb-userguide.pdf). Physical inactivity was defined according to a non-confirmatory response to the following question: “During the past month, other than your regular job, did you participate in any physical activities or exercises such as running, calisthenics, golf, gardening, or walking for exercise?”

*** Respondents rated their general health as being excellent, very good, good, fair, or poor. The responses were then categorized into two groups: 1) those who reported that their health was excellent, very good, or good and 2) those who reported that their health was fair or poor. Fair or poor health status was defined as a report of fair or poor health status. All respondents were asked to determine how many days during the past 30 days their mental health status (e.g., stress, depression, and problems with emotions) was not good. The respondents were divided into two groups: those who reported frequent mental distress (≥14 mentally unhealthy days during the past 30 days) and those who reported no frequent mental distress (<14 mentally unhealthy days during the past 30 days). Frequent mental distress was defined as a report of ≥14 mentally unhealthy days during the past 30 days. Overweight or having obesity was defined as a body mass index ≥25 kg/m^2^, or ≥30 kg/m^2^, respectively, calculated from self-reported weight and height. Coronary heart disease was defined as having ever been told by a doctor, nurse, or other health care professional that the respondent had a heart attack (myocardial infarction) or angina. Asthma was defined as having ever been told by a doctor, nurse, or other health care professional that the respondent had asthma and still had it at the time of survey participation. Chronic obstructive pulmonary disease was defined as having ever been told by a doctor, nurse, or other health care professional that the respondent had chronic obstructive pulmonary disease, emphysema, or chronic bronchitis. Diabetes was defined as having ever been told by a doctor, nurse, or other health care professional that the respondent had diabetes, excluding gestational diabetes, prediabetes, or borderline diabetes. Arthritis was defined as having ever been told by a doctor, nurse, or other health care professional that the respondent had some form of arthritis, rheumatoid arthritis, gout, lupus, or fibromyalgia. High blood pressure was defined as having ever been told by a doctor, nurse, or other health care professional the respondent had high blood pressure. High blood cholesterol was defined as having ever been told by a doctor, nurse, or other health care professional that the respondent’s blood cholesterol was high. Depression was defined as having ever been told by a doctor, nurse, or other health care professional that the respondent had a depressive disorder, which includes depression, major depression, dysthymia, or minor depression.

††† https://www.ihs.gov/forpatients/.

§§§ https://azdhs.gov/documents/operations/managing-excellence/azhip.pdf.

¶¶¶ https://www.cdc.gov/chronicdisease/tribal/tribalpractices.htm.

**** https://www.cdc.gov/chronicdisease/tribal/factsheet.htm.
